# Fresh Parent’s Own Milk for Preterm Infants: Barriers and Future Opportunities

**DOI:** 10.3390/nu16030362

**Published:** 2024-01-26

**Authors:** Carrie-Ellen Briere, Jessica Gomez

**Affiliations:** 1 Elaine Marieb College of Nursing, University of Massachusetts Amherst, Amherst, MA 01003, USA; 2 Institute of Nursing Research and Evidence-Based Practice, Connecticut Children’s, Hartford, CT 06106, USA; 3 Department of Pediatrics/Neonatal-Perinatal Medicine, McGovern School of Medicine, The University of Texas Health Science Center at Houston, Houston, TX 77030, USA; jessica.gomez@uth.tmc.edu

**Keywords:** human milk, breastfeeding, breast milk, cell, bioactive, newborn nutrition, neonatal, NICU

## Abstract

While direct at-the-breast feeding is biologically optimal, Neonatal Intensive Care Unit (NICU) admission due to infant immaturity or illness often necessitates the expression and storage of parent’s milk. The provision of freshly expressed (never stored) parent’s own milk to preterm infants is not widely prioritized, and this article provides an exploration of NICU practices and their implications for feeding premature or ill infants with parent’s own milk. In this article, we discuss the potential biological benefits of fresh parent’s own milk, highlighting its dynamic components and the changes incurred during storage. Research suggests that fresh milk may offer health advantages over stored milk. The authors advocate for further research, emphasizing the need for standardized definitions. Research is needed on the biological impact of fresh milk, both short- and long-term, as well as defining and understanding healthcare economics when using fresh milk.

## 1. Introduction and Overview of NICU Practices and Implications for Parent’s Own Milk Feeding

Direct at-the-breast feeding provides the biological norm of infant feeding, yet admission to a Neonatal Intensive Care Unit (NICU) creates barriers that ultimately alter the delivery of this biologically optimized, fresh parent’s own milk. Many infants admitted to an NICU cannot be orally fed at some point during their hospitalization. The inability to feed orally may be related to their immaturity, diagnosis, or illness severity, which may limit oral or enteral feeding. Parents who want to provide human milk for their premature or ill newborns must initiate and maintain their milk supply by expressing milk. For infants who cannot be fed orally, this expressed parent’s milk is fed through a feeding tube or stored in a refrigerator or freezer.

Within the last decade, it has become increasingly common for Neonatal Intensive Care Units (NICUs) to have dedicated rooms and personnel to prepare enteral feedings. These milk labs or milk preparation rooms are where formula and human milk (parent’s own or donor) are prepared with ordered additives or fortifications and dispensed into individual feeding containers. Initially, many of these rooms were focused solely on mixing and preparing formula due to clinical practice recommendations based on research that showed these centralized and sterile environments reduced formula contamination [1,2]. However, more NICUs have been preparing and dispensing both formula and human milk feedings from a central milk preparation room in recent years [3,4]. In addition to the goal of preventing contamination, other reasons for creating these rooms have been to initiate individualized, targeted fortification of human milk [3], and overall cost savings from a centralized process [5].

Historically, human milk handling and preparation have occurred at the bedside where clinical staff (typically nurses) receive the milk, divide it into storage containers, label it, and then determine storage outcome (refrigerator or freezer) based on infant feeding needs. While there may be benefits to centralizing human milk preparation away from the bedside and direct clinical care providers, it is essential to know that these processes also may make it less likely that infants will receive freshly expressed parent’s milk. Prepared human milk feedings from milk preparation rooms are often prepared at set times during the day and may not account for parents who will provide expressed milk at a specific time, and due to this, freshly pumped parent’s milk will then be refrigerated or frozen for later use. Along with other barriers, barcoded feeding management tools now require specific barcodes and labeling systems on every container of expressed milk before feeding to an infant. These systems reduce potential misadministration but can also create an additional barrier in the clinical care workflow to feeding freshly expressed parent’s milk. 

**Figure 1:**
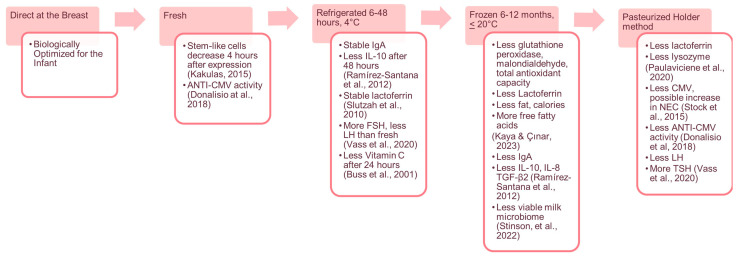
Hierarchy of human milk and changes in components [12,14,15,16,17,18,19,20,21,22]. Legend: IgA = Immunoglobulin A; IL-10 = Interleukin-10: TGF-β2 = Transforming growth factor; CMV = Cytomegalovirus; NEC = Necrotizing enterocolitis; LH = Luteinizing hormone; TSH = Thyroid stimulating factor: FSH = follicle stimulating hormone.

While the receipt of fresh parent’s own milk in some countries is challenging due to barriers such as hospital handling practices and maternal availability, other countries do not feed fresh milk due to fear of infection from viruses such as Cytomegalovirus (CMV). In these hospitals, freshly expressed parent’s milk is commonly pasteurized prior to infant feeding to inactivate potentially harmful viruses [6,7]. While macronutrients are not impacted much through pasteurization, there is some evidence that they may reduce preterm infant fat absorption [8]. Additionally, heat treatment and cold storage impact many bioactive components of fresh human milk [9,10,11]; see Figure 1. Therefore, it is vital to recognize and consider the potential biological benefits of fresh parent’s own milk on infant health and development. In this paper, we define fresh milk as expressed parent’s own milk fed to an infant within four hours of expression, as this is when cell components begin to degrade [12] and has been used in other studies [6,13].

## 2. Potential Benefits of Fresh Parent’s Own Milk to Infant Health and Development

While once thought of as solely a nutrition source full of immunological support, research on human milk has transformed our understanding of this incredible, biologically complex fluid. In addition to the main building blocks of carbohydrates, proteins, fat, minerals, and vitamins, scientists have started to explore the biological importance of milk’s other components. These include cells (both human and bacterial), cellular components (including extracellular vesicles, exosomes, miRNA), growth factors, digestive enzymes, hormones, cytokines, and transporters [23,24,25]. 

Human milk is a complex biological fluid filled with ever-changing components. Biologically, human milk is intended to be fed directly at the breast. When human milk is expressed for later feeding, it is important to consider that the bioactive components will ultimately change due to biological activity, time, and environment (e.g., refrigeration, freezing, type of storage container). There is a paucity of research and knowledge about these changes and how they may impact infant health [26]. However, we do know that in term infants, researchers have found that compared to feeding stored expressed milk, direct-at-the-breast feeding is more protective against otitis media [27], coughing and wheezing episodes [28], and asthma [29,30]. Additionally, when considering fresh parent’s own milk compared to pasteurized parent’s own milk, a group of researchers found that the risk of bronchopulmonary dysplasia was reduced in infants who received fresh parent’s own milk [31]. 

There is not yet evidence on the exact mechanisms which may be responsible for varying health impacts with fresh versus stored milk. However, it can be hypothesized that the protective factors of milk components are diminished when components are reduced or eliminated through storage and feeding practices. For example, adrenomedullin (AM) is a hormone in the gastrointestinal (GI) tract present in human milk [32]. Within the GI tract, AM is known to help regulate many physiological processes, including anti-inflammation, organ protection, tissue repair, and improved intestinal barrier function [32]. Compared to human milk from parents of full-term infants, AM levels are higher in preterm milk [33]. Pelia et al. found that after 96 h of refrigeration, only 2% concentration of AM remained compared to its freshly pumped levels (*p* < 0.05) [33]. Considering the known role of AM in modulating impacts from hypoxia and inflammation, a substantial change in this peptide hormones availability in preterm milk could have significant health implications. 

A recent systematic review reported that in addition to some changes in macronutrient content with freezing, there are significant decreases in the amounts of glutathione peroxidase, antioxidant capacity, and lactoferrin [14]—all of which are believed to provide beneficial developmental support and protection within human milk. Lactoferrin in human milk is one of the mechanisms that help decrease the risk of necrotizing enterocolitis (NEC) in preterm infants [34]. Moreover, freezing milk reduces the milk pH and the total bacterial colony count, affecting probiotic bacteria with unknown consequences for preterm infants [35,36]. 

Another component of human milk is living cells [24,25]. A research team has recently found that there may be an active intercellular communication system within human milk [37]. In this research study, the authors show that cells of freshly pumped human milk have intercellular connections, many different cell-to-cell contact regions, and thin pseudopods (cellular regions used to aid cell movement [38]). More research is needed to understand what this potential intercellular communication network may mean from a biological perspective. Many cells in human milk are believed to be alive after initial expression; however, the cells will eventually die after time and storage. No known work has yet examined cell death processes in human milk, but we hypothesize that cell death from storage length and condition may follow the pathway of necrosis, which is an uncontrolled death induced by an external injury, such as heating or freezing [39]. When cells die and rupture, the contents are released into the human milk, some of which may activate an inflammatory response [40]. 

In the last few years, research protocols and preliminary results have been published measuring the impact of receipt of freshly expressed (within 4 h) human milk on premature infant health [13]. Intervention infants were fed fresh milk 1× per day until 32 weeks gestational age and were compared to control infants (fed only frozen human milk—parent’s or donors’). This research team found that infants who received freshly expressed parent’s own milk once a day had significantly fewer days of total parenteral nutrition (*p* < 0.01) and a shorter duration of mechanical ventilation. Despite preliminary promising results, the study was underpowered, and it is unclear if confounders were used to control outcomes related to mechanical ventilation and respiratory outcomes. For example, the intervention group had better respiratory outcomes but received significantly more antenatal steroids than the control group—50% vs. 12%, *p* < 0.01. A different research team in China has started to look at the health and developmental impacts of feeding fresh parent’s own milk (defined as expressed within 3 h of feeding) compared to pasteurized parent’s own milk to preterm infants < 1500 g [41]. After adjusting for confounders, the fresh parent’s own milk group had a higher survival rate without severe complications (*p* = 0.014) and lower bronchopulmonary dysplasia rates (*p* = 0.010). Receipt of fresh parent’s own milk led to faster regaining of birthweight (*p* = 0.021), reaching full enteral feeding sooner (*p* = 0.024), and receiving less parenteral nutrition (*p* = 0.045). Both studies provide initial insight into the building evidence that types of human milk feeding may provide varying levels of protection and developmental support for preterm infants. 

One of the challenges with research on human milk feeding and understanding overall benefits and importance is that human milk feeding is not universally recorded in the clinical record with specifics on the type of human milk. Many hospitals and reporting agencies group all types of human milk (direct-at-breast, freshly pumped, refrigerated, frozen, pasteurized own parent’s milk, pasteurized donor human milk), making it difficult to fully understand how the type of human milk feeding impacts infant health. In our pursuit of further understanding of the importance of fresh parent’s own milk, an additional challenge persists with the definition of fresh milk ranging from within three hours of pumping and left at room temperature [41] to any type of pumped milk within 48 h stored in the refrigerator [42].

## 3. Future Research and Directions

Current practices in NICUs across the United States do not consistently prioritize fresh milk despite the potential benefit of higher concentrations of bioactive ingredients. Therefore, understanding the healthcare costs or savings related to feeding fresh versus frozen parent’s own milk is needed. Johnson et al. demonstrated that an increase in parent’s own milk in the first two weeks of life was associated with a lower healthcare cost demonstrated by 0.26 fewer hospitalizations (*p* = 0.04) in the first year of life, fewer subspecialists required (*p* = 0.04), and fewer ongoing therapies (*p* = 0.04) used at two years [43]. Future research should replicate this study but define the amount of fresh or frozen parent’s own milk the infant receives and follow healthcare use through the first two years of life.

Sun et al. [13] and Huang et al. [41] demonstrated the safety, feasibility, and potential benefits of feeding fresh parent’s own milk at least once daily. Sun et al. found that 87.5% of the intervention group (*n* = 98) provided at least one fresh feed daily and had a lower duration of mechanical ventilation and total parenteral nutrition (2019). Huang et al. found that at least one fresh feed each day was associated with a higher survival rate without severe complications (*p* = 0.014) and a lower incidence of BPD (*p* = 0.010) after adjusting for confounders [41]. Neither of these studies was appropriately powered to measure changes in NEC, Length of Stay, or Retinopathy of Prematurity. However, Sun et al. have published a protocol for a large nationwide randomized controlled trial to provide sufficient power to answer these questions [6]. 

Many investigators have demonstrated measurable biological changes related to infant diet in preterm infants through non-invasive oxidative stress measures, changes in the microbiome [44], or changes in secretion of immunoglobulin A (sIgA) levels in body fluids [45]. Further research is needed to understand ways to quantify biological changes in preterm infants related to fresh versus frozen parent’s own milk to identify short- or long-term health benefits if they exist.

## 4. What Can We Do Today?

Feeding all fresh milk may not be feasible as preterm infants cannot take large volume feeds for the first few weeks or months of life. For this reason, parent’s own milk is frozen until it is needed. Freezing was historically performed to decrease the potential load of CMV in the milk, but a study by Lanzieri et al. found that in a meta-analysis of 17 studies of very low birth weight infants, sepsis-like syndrome occurred at similar rates between fresh and frozen parent’s own milk (1.4% vs. 1.7%, respectively) [46]. While shedding of CMV occurs in approximately 90% of seropositive women [47], postnatally acquired CMV only occurs in preterm infants from CMV-positive milk at rates of around 6.5% with approximately 0.7–2.4% developing a sepsis-like syndrome [46]. 

Additionally, the long-term cognitive effects of postnatally acquired CMV are unclear. Brecht et al., in a sample of 42 adolescents born at 23–32 weeks gestation (*n* = 19 postnatally CMV+), concluded that postnatally acquired CMV may negatively affect cognitive function [48]. However, the sample by Brecht et al. was not adequately powered, and they could not control for maternal education, which was lower in the postnatally acquired CMV group. Conversely, in a cohort of 356 infants born at < 32 weeks gestation (n = 49 postnatally CMV+), the Bayley Test scores showed no difference at six years of age [48].

Therefore, it may be reasonable to promote freshly expressed parent’s own milk for the diet of preterm and ill infants in the NICU. Of note, the American Academy of Pediatrics mentioned the prioritization of fresh milk for preterm infants in their 2012 policy statement [49]; however, in their most recent update [50], that statement is omitted without explanation. 

While there are many barriers to achieving fresh human milk feeding based on parental circumstances and hospital practices, it is essential to note that the few studies that have been conducted have found health benefits with as little as one fresh feeding per day [13,41]. Collaborative discussions with families about the potential benefits of fresh parent’s own milk can empower them to recognize that their milk is a personalized medicine for their infant [51] and encourage parents to prioritize pumping at the bedside as often as possible to provide fresh parent’s own milk. In the future, more work is needed to address disparities in the ability of all families to be present in the NICU independent of other factors (e.g., economic, family, social).

To provide fresh parent’s own milk to preterm infants, NICU providers can prioritize and promote bedside pumping, early oral care with colostrum, and direct breastfeeding as soon as appropriate. Current practice recommendations for pump-dependent mothers in the NICU promote frequent pumping without regard to the location [52]. Pumping at the infant’s bedside in the NICU would provide easy access to freshly pumped milk, and qualitative studies have shown that mothers pumping near their infant promoted feelings of emotional closeness and produced positive feelings toward pumping [53,54]. Moreover, pumping near their infant may improve milk output [55].

Early oral care with colostrum is a low-cost, low-risk intervention that may improve preterm infant morbidities like NEC and late-onset sepsis [56] and help establish a healthy oral microbiome [44]. Oral care with colostrum is 0.2 mL of early milk expressed in the first hours to 5 days of life that is instilled into the buccal cavity using a tuberculin syringe [57]. Early and frequent milk expression, as with colostrum harvesting, is linked to an increased milk supply for pump-dependent mothers [58,59] and is a feasible way to increase access to fresh parent’s own milk.

The support and promotion of direct breastfeeding for mothers of preterm infants improved the length of breastfeeding [60] and maternal breastfeeding self-efficacy [61]. Direct breastfeeding has the added benefits of supporting normal jaw development and less risk of malocclusion [62]. Bedside pumping, early oral care with colostrum, and direct breastfeeding all promote fresh milk and may increase breastfeeding duration and, therefore, improve preterm infant health outcomes [63]. 

NICUs promoting fresh milk can also educate families about the difference between fresh and stored milk. RUSH University’s NICU provides an example of educational material teaching families the difference between fresh and stored milk [64]. Including families in decisions about their infant can improve their sense of shared decision-making and parental autonomy [65].

## 5. Summary

Despite the increasing prevalence of dedicated milk preparation rooms in NICUs aimed at reducing contamination and streamlining the feeding process, these centralized environments often result in freshly expressed parent’s milk being refrigerated or frozen for later use. Barriers such as set preparation times, barcoded feeding management tools, and concerns over potential CMV infection in some countries have further complicated the availability and delivery of fresh milk to infants. Nevertheless, the unique benefits that biologically optimized, fresh human milk offers to premature or ill newborns cannot be overlooked.

A growing body of evidence indicates potential biological advantages for freshly pumped parent’s own milk compared to refrigerated/frozen parent’s own milk. This paper advocates for collaborative discussions with families and NICU providers, prioritizing bedside pumping, early oral care with colostrum, and direct breastfeeding to increase access to fresh milk. Furthermore, we call for ongoing research to comprehensively evaluate the benefits and costs of prioritizing fresh human milk in the NICU. 

Looking forward, the next steps in this area of research should include the following:

- Standardized Definitions: Develop standardized definitions and classifications of different types of human milk feeding (e.g., fresh, refrigerated, frozen) to facilitate consistent reporting and analysis in clinical care and research.
- Healthcare Economics: Investigate the healthcare cost implications of feeding fresh human milk versus frozen milk, considering short-term and long-term outcomes, which may inform policy decisions.
- Biological Impact: Continue to explore the biological changes in human milk over time and during storage and assess how these changes impact the health and development of preterm infants.
- Long-Term Outcomes: Conduct long-term follow-up studies to understand the potential lifelong benefits of fresh milk feeding on preterm infants’ health, growth, and neurodevelopment.
- Parental Education and Support: Develop and implement educational programs to inform parents about the benefits of fresh milk and empower them to participate in the decision-making process regarding their infant’s nutrition.

This work is needed to continue to build a foundation of knowledge for reevaluating current practices and promoting fresh milk usage to ultimately enhance the care and outcomes of our most fragile infants in NICUs.
